# A Practical Treatise on Urinary and Renal Diseases

**Published:** 1885-08

**Authors:** 


**A Practical Treatise on Urinary and Renal Diseases,** including Urinary Deposits, illustrated by numerous cases and engravings, by William Roberts, M. D., F. R. S., Prof. of Medicine, Victoria University, assisted by Robert McGuire, M.D., London, M. R. C. P., Physician to St. Mary's Hospital. Fourth edition. Philadelphia: Lea Brothers & Co. 1885.

We have looked forward with interest to the appearance of a new edition of this book. Although of late years many new books have been published upon the subject, none have supplanted Roberts in our estimation. To those who are not familiar with it, we would say that the design of the book is to give an account of the organic diseases of the kidney, and of those diseases and disorders of which the chief characteristic is some alteration of the urine.

The work is naturally divided into three parts. First, a consideration of the physical and chemical properties of the urine, and to the various alterations which it undergoes in health and disease. In this particular department, the work of Neubauer and Vogel is complete and exhaustive, but because it is so, it is not convenient for the general practitioner. The work before us separates and selects, from the vast array of chemical and physiological researches on this subject, those facts and that knowledge which have a direct clinical value.

The second part is devoted to the consideration of diabetes, gravel and calculus, and chylous urine, and here we find many additions to the previous edition, and the subject is brought up to the level of our existing knowledge. The organic diseases of the kidney form the subject of the third and last part of the work. The most important of these, Bright's Diseases are treated with a fullness commensurate with their gravity and importance. The chapter on albuminuria has been almost entirely re-written, and we find much entirely new matter in this part of the work. Throughout the book we are glad to see that the alterations and additions have been in the line of practical work. Non-established theories are not dwelt upon, and that only has been introduced which will tend to enhance its clinical value. We cordially commend the present edition as one of the best works on its special subject, and one which every practitioner should have.

